# Generic docetaxel chemotherapy induced skin toxicities in breast cancer patient

**DOI:** 10.11604/pamj.2016.24.164.9908

**Published:** 2016-06-27

**Authors:** Iliass Elalami, Mohamed Ichou

**Affiliations:** 1 Departments of Medical Oncology, Military Hospital Med V, Rabat, Morocco

**Keywords:** Generic docetaxel, skin toxicities, breast cancer

## Image in medicine

Female patient, 52 years old, three months after mastectomy due to breast cancer was subjected to chemotherapy with docetaxel. After the first cycle she presented erythema and dysesthesia of the burning sensation type that greatly improved in 2 weeks. After the next session there was relapse of symptoms. She was treated with a topical corticosteroid for 7 days. There was partial improvement of symptoms. At each new chemotherapy session she presented the same symptoms with greater intensity and less expressive improvement with the treatment. Docetaxel belongs to the taxane group and act by inhibiting mitotic activity due to the suppression of microtubule depolymerization. Signs of dermatological toxicity are observed in around 65% of cases and include alopecia, hypersensitivity reactions and ungual alterations. The reactions usually occur after the first treatment cycle and are dose dependent, with relief of discomfort during relapses when the amount of the drug being given is decreased. There usually is spontaneous resolution after 2 weeks, with recurrence when the drug is reintroduced. The use of topical or systemic corticosteroids and application of cold compresses is recommended for incapacitating pain. Preventive treatment with pyridoxine was reported as beneficial in one study. It is recommended to apply local hypothermia to acral regions during medication infusion to decrease local drug perfusion. A few studies suggests that some toxic effects of docetaxel may be related to the excipients used in different formulations of the drug.

**Figure 1:**
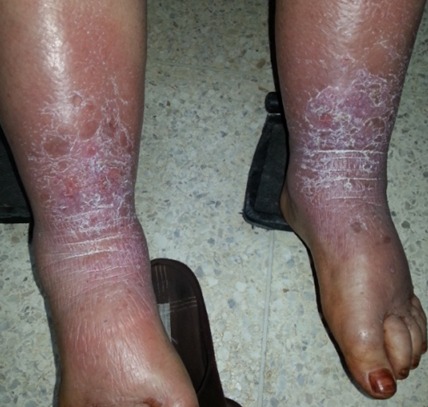
Presence of erythema with profound desquamation and skin edema

